# Crystal structure and Hirshfeld surface analysis of 2-amino-6-[(1-phenyl­eth­yl)amino]-4-(thio­phen-2-yl)pyridine-3,5-dicarbo­nitrile

**DOI:** 10.1107/S2056989023003845

**Published:** 2023-05-05

**Authors:** Farid N. Naghiyev, Victor N. Khrustalev, Khammed A. Asadov, Mehmet Akkurt, Ali N. Khalilov, Ajaya Bhattarai, İbrahim G. Mamedov

**Affiliations:** a Department of Chemistry, Baku State University, Z. Khalilov str. 23, Az, 1148, Baku, Azerbaijan; b Peoples’ Friendship University of Russia (RUDN University), Miklukho-Maklay St. 6, Moscow, 117198, Russian Federation; c N. D. Zelinsky Institute of Organic Chemistry RAS, Leninsky Prosp. 47, Moscow, 119991, Russian Federation; d Department of Physics, Faculty of Sciences, Erciyes University, 38039 Kayseri, Türkiye; e "Composite Materials" Scientific Research Center, Azerbaijan State Economic University (UNEC), H. Aliyev str. 135, Az 1063, Baku, Azerbaijan; f Department of Chemistry, M.M.A.M.C (Tribhuvan University) Biratnagar, Nepal; Katholieke Universiteit Leuven, Belgium

**Keywords:** crystal structure, pyridine ring, thio­phene ring, disorder, Hirshfeld surface analysis

## Abstract

In the title compound, C_19_H_15_N_5_S, the thio­phene ring is disordered in a 0.6:0.4 ratio by an approximate 180° rotation of the ring around the C—C bond linking it to the pyridine ring. In the crystal, the mol­ecules are linked by N—H⋯N hydrogen bonds into dimers with an *R* ^2^_2_(12) motif, forming chains along the *b*-axis direction. These chains are connected to each other by further N—H⋯N hydrogen bonds, forming a three-dimensional network. Furthermore, N—H⋯π and π–π [centroid–centroid separations = 3.899 (8) and 3.7938 (12) Å] inter­actions also contribute to the crystal cohesion. A Hirshfeld surface analysis indicated that the most important contributions to the surface contacts are from H⋯H (46.1%), N⋯H/H⋯N (20.4%) and C⋯H/H⋯C (17.4%) inter­actions.

## Abstract 2

In the crystal, the mol­ecules are connected by N—H⋯N hydrogen bonds into dimers with an 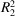 (12) motif, forming chains along the *b*-axis direction. These chains are linked to each other by N—H⋯N hydrogen bonds, N–H⋯π and π–π inter­actions, forming a three-dimensional network.

## 1. Chemical context

Diverse C—C, C—N and C—O bond-formation methods play important roles in organic synthesis. The reaction scopes have also been greatly expanded, employing these methods in different fields of chemistry, in both academia and industry (Çelik *et al.*, 2023 ▸; Chalkha *et al.*, 2023 ▸; Tapera *et al.*, 2022 ▸; Gurbanov *et al.*, 2020 ▸; Zubkov *et al.*, 2018 ▸). The pyridine moiety is a widespread structural motif that can be found in various natural products and pharmacologically active compounds. 3,5-Di­cyano­pyridines have been reported as inter­mediates in the synthesis of pyrido[2,3-*d*]pyrimidines, pyridothienotriazines, aza­benzanthracenes and pyrimidine *S*-nucleoside derivatives with a broad spectrum of biological activity (Cocco *et al.*, 2005 ▸; Zhang *et al.*, 2022 ▸; Poustforoosh *et al.*, 2022 ▸). The design of new 3,5-di­cyano­pyridine derivatives is thus of great inter­est. 
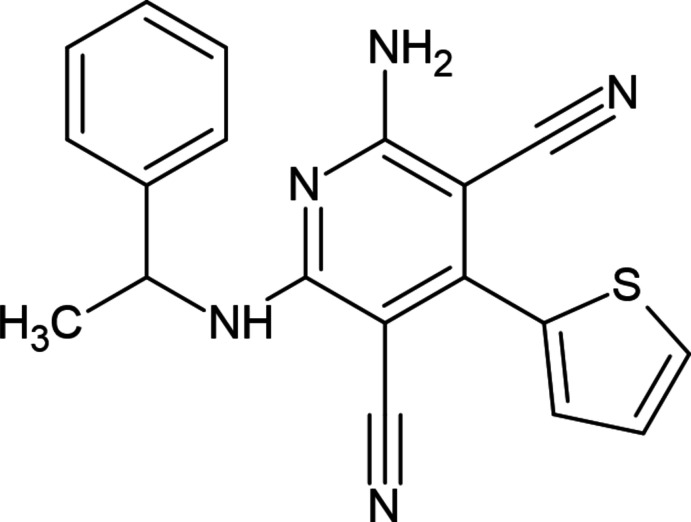


Continuing our studies of pyridine derivatives exhibiting biological activity, we designed and synthesized a novel 3,5-di­cyano­pyridine in this series. Thus, in the framework of our ongoing structural studies (Naghiyev *et al.*, 2020 ▸, 2021 ▸, 2022 ▸), we report the crystal structure and Hirshfeld surface analysis of the title compound, 2-amino-6-[(1-phenyl­eth­yl)amino]-4-(thio­phen-2-yl)pyridine-3,5-dicarb­o­nitrile.

## 2. Structural commentary

**Figure 1:**
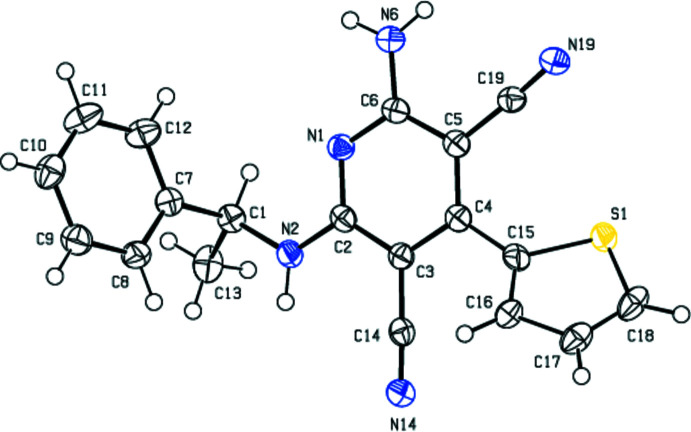
The mol­ecular structure of the title compound, showing the atom labelling and displacement ellipsoids drawn at the 30% probability level. For clarity, the minor disorder component is not shown.

The pyridine ring (N1/C2–C6) of the title compound (Fig. 1 ▸) is largely planar [maximum deviation = 0.015 (2) Å for C5]. The thio­phene and 1-phenyl­ethan-1-amine groups are linked to the central pyridine ring in an equatorial arrangement. The major and minor parts (S1/C15–C18 and S1*A*/C15*A*–C18*A*) of the disordered thio­phene ring make dihedral angles of 44.8 (5) and 48.9 (6)°, respectively, with the pyridine ring. The dihedral angle between the phenyl (C7–C12) and pyridine (N1/C2–C6) rings is 64.42 (11) °.

## 3. Supra­molecular features and Hirshfeld surface analysis

**Figure 2:**
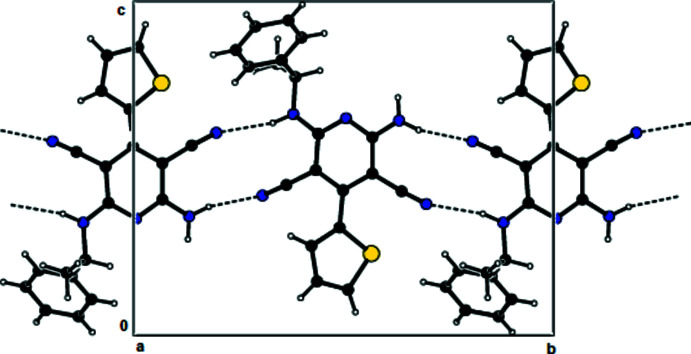
View of the mol­ecular packing along the *a* axis. N—H⋯N hydrogen bonds are shown as dashed lines. For clarity, the minor disorder component is not shown.

**Figure 3:**
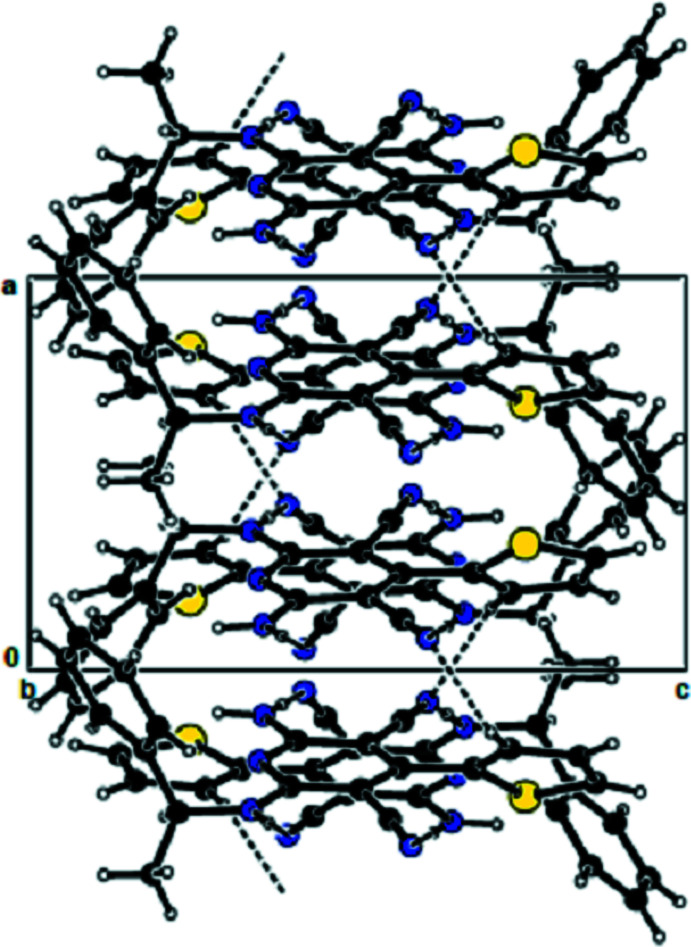
View of the mol­ecular packing along the *b* axis. Hydrogen bonds are shown as dashed lines.

**Figure 5:**
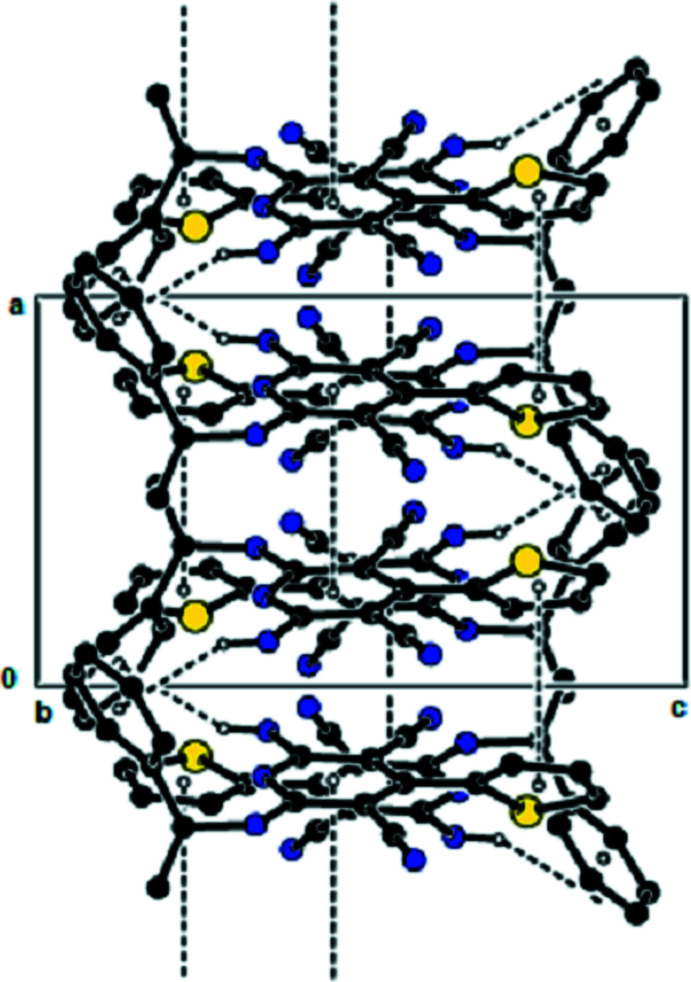
View of the mol­ecular packing along the *b* axis. N—H⋯π inter­actions and π–π stacking inter­actions are shown as dashed lines.

In the crystal, the mol­ecules are linked by N—H⋯N hydrogen bonds into dimers with an 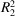 (12) motif (Bernstein *et al.*, 1995 ▸; Table 1 ▸, Fig. 2 ▸), forming chains along the *b*-axis direction. These chains are connected to each other by further N—H⋯N hydrogen bonds, forming a three-dimensional network (Tables 1 ▸ and 2 ▸, Figs. 3 ▸ and 4 ▸). Furthermore, N—H⋯π and π–π inter­actions [*Cg*1⋯*Cg*1^i^ = 3.899 (8) Å; slippage = 1.899 Å; *Cg*3⋯*Cg*3^ii^ = 3.7938 (12) Å; slippage = 1.383 Å; symmetry codes: (i) −*x*, 1 − *y*, *z*; (ii) 1 − *x*, 1 − *y*, *z*; *Cg*1 and *Cg*3 are the centroids of the major component of the disordered thio­phene ring and of the pyridine ring, respectively] also contribute to crystal cohesion (Figs. 5 ▸ and 6 ▸).

**Figure 7:**
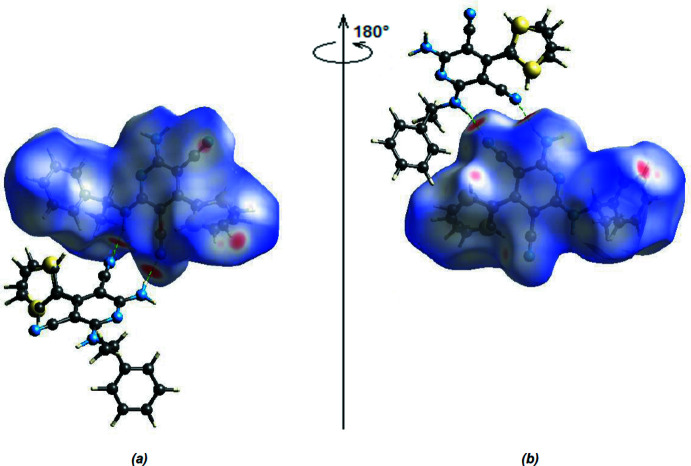
(*a*) Front and (*b*) back sides of the three-dimensional Hirshfeld surface of the title compound mapped over *d*_norm_, with a fixed colour scale of −0.4485 to +1.5784 a.u. N—H⋯N hydrogen bonds are shown as dashed lines.

**Figure 8:**
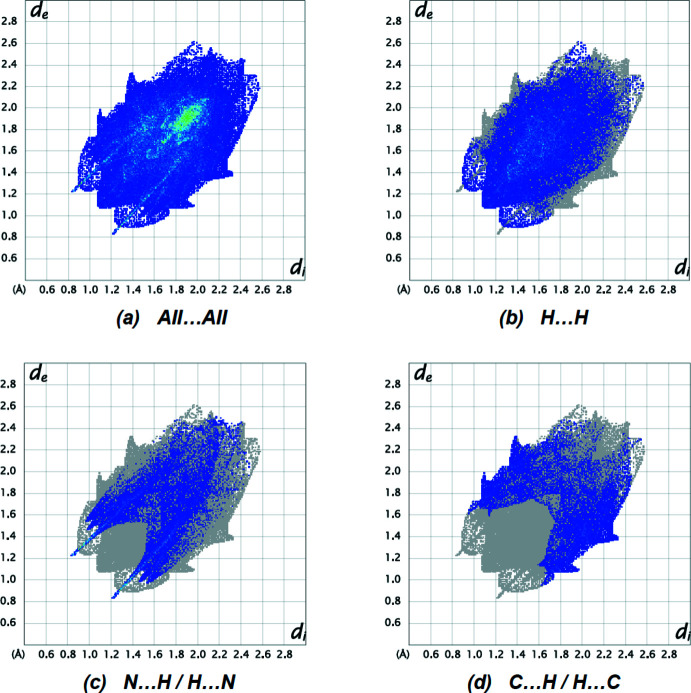
The two-dimensional fingerprint plots of the title compound, showing (*a*) all inter­actions, and delineated into (*b*) H⋯H, (*c*) N⋯H/H⋯N and (*d*) C⋯H/H⋯C inter­actions. [*d*_e_ and *d*_i_ represent the distances from a point on the Hirshfeld surface to the nearest atoms outside (external) and inside (inter­nal) the surface, respectively].

*Crystal Explorer 17.5* (Spackman *et al.*, 2021 ▸) was used to generate Hirshfeld surfaces and two-dimensional fingerprint plots in order to qu­antify the inter­molecular inter­actions in the crystal. The inter­molecular inter­actions are depicted as red spots, which denotes the N—H⋯N hydrogen bonds, on the Hirshfeld surface mapped over *d*_norm_ in the range −0.4485 to +1.5784 a.u. (Fig. 7 ▸ *a*,*b*). Fig. 8 ▸ shows the two-dimensional fingerprint plots. The H⋯H contacts comprise 46.1% of the total inter­actions. Besides this contact, N⋯H/H⋯N (20.4%) and C⋯H/H⋯C (17.4%) inter­actions make significant contributions to the total Hirshfeld surface. The percentage contributions of the C⋯C, N⋯C/C⋯N, N⋯N, S⋯C/C⋯S, S⋯H/H⋯S and S⋯S contacts are 6.9, 3.8, 2.7, 1.5, 0.6 and 0.6%, respectively.

## 4. Database survey

The four related compounds found as a result of the search for ‘2,6-di­amino-4-(thio­phen-2-yl)pyridine-3,5-dicarbo­nitrile’ in the Cambridge Structure Database (CSD, Version 5.42, update of September 2021; Groom *et al.*, 2016 ▸) are MUCLAA (Vu Quoc *et al.*, 2019 ▸), WOJCIJ (Vishnupriya *et al.*, 2014*a* ▸), WOPLAQ (Vishnupriya *et al.*, 2014*b* ▸) and DOPWOW (Vishnupriya *et al.*, 2014*c* ▸).

In the crystal of MUCLAA (space group *P*2_1_/*c*), chains running along the *b*-axis direction are formed through N—H⋯O inter­actions between the 1,4-di­hydro­pyridine N atom and one of the O atoms of the ester groups. Neighbouring chains are linked by C—H⋯O and C—H⋯π inter­actions. In the crystal of WOJCIJ (space group *P*2_1_/*c*), inversion dimers linked by pairs of N—H⋯N hydrogen bonds generate 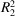 (16) loops and the dimers are linked by C—H⋯π and aromatic π–π stacking inter­actions into a three-dimensional network. In WOPLAQ (space group *P*2_1_/*n*), inversion dimers linked by pairs of N—H⋯N_c_ (c = cyanide) hydrogen bonds generate 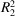 (16) loops. In DOPWOW (space group *Pbca*), inversion dimers linked by pairs of N—H⋯N_n_ (n = nitrile) hydrogen bonds generate 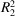 (16) loops. Aromatic π–π stacking and very weak C—H⋯π inter­actions are also observed.

## 5. Synthesis and crystallization

To a solution of 2-(thio­phen-2-yl­methyl­ene)malono­nitrile (0.82 g; 5.1 mmol) and malono­nitrile (0.34 g; 5.2 mmol) in methanol (25 mL), phenyl­ethyl­amine (0.63 g; 5.2 mmol) was added and the mixture was stirred at room temperature for 48 h. Then 15 mL of methanol were removed from the reaction mixture, which was left overnight. The precipitated crystals were separated by filtration and recrystallized from ethanol/water (1:1) solution (yield 94%; m.p. 460–461 K).

^1^H NMR (300 MHz, DMSO-*d*_6_, ppm): 1.55 (*d*, 3H, CH_3_, ^3^ *J*_H–H_ = 7 MHz); 5.45 (*k*, 1H, CH—Ar, ^3^ *J*_H–H_ =7,1 MHz); 7.21–7.88 (*m*, 11H, 5CH_arom_ + 3CH_thien­yl_ + NH_2_ + NH); ^13^C NMR (75 MHz, DMSO-*d*_6_, ppm): 21.69 (CH_3_), 50.00 (CH—Ar), 79.77 (=C_tert_), 80.92 (=C_tert_), 116.85 (CN), 116.97 (CN), 127.14 (2CH_arom_), 127.22 (CH_arom_), 128.11 (CH_thien­yl_), 128.63 (2CH_arom_), 130.14 (CH_thien­yl_), 130.75 (CH_thien­yl_), 134.53 (C_ar_), 144.53 (C_thien­yl_), 152.30 (=C_tert_), 158.70 (N=C_tert_), 161.38 (=C_tert_).

## 6. Refinement

Crystal data, data collection and structure refinement details are summarized in Table 3 ▸. The thio­phene ring in the title compound was modelled as disordered over two sets of sites related by an approximate rotation of 180° about the C4—C15 bond in a 0.6:0.4 ratio. EADP commands in *SHELXL* were used for the *U*_ij_ values of equivalent atom pairs (*e.g*., C16 and C16*A*) and DFIX commands were used to restrain the nearest-neighbour and next-nearest-neighbour bond distances in the two disorder components to be equal with a standard deviation of 0.03 Å. All C-bound H atoms were placed in calculated positions (0.95–1.00 Å) and refined as riding with *U*_iso_(H) = 1.2 or 1.5*U*_eq_(C). The N-bound H atoms were located in a difference map and refined with *U*_iso_(H) = 1.2*U*_eq_(N) [N2—H2 = 0.91 (3) Å, N6—H6*A* = 0.91 (3) Å, N6—H6*B* = 0.89 (3) Å].

## Supplementary Material

Crystal structure: contains datablock(s) I, global. DOI: 10.1107/S2056989023003845/vm2282sup1.cif

Structure factors: contains datablock(s) I. DOI: 10.1107/S2056989023003845/vm2282Isup2.hkl

Supporting information file. DOI: 10.1107/S2056989023003845/vm2282Isup3.cml

CCDC reference: 2260011

Additional supporting information:  crystallographic information; 3D view; checkCIF report

## Figures and Tables

**Figure 4:**
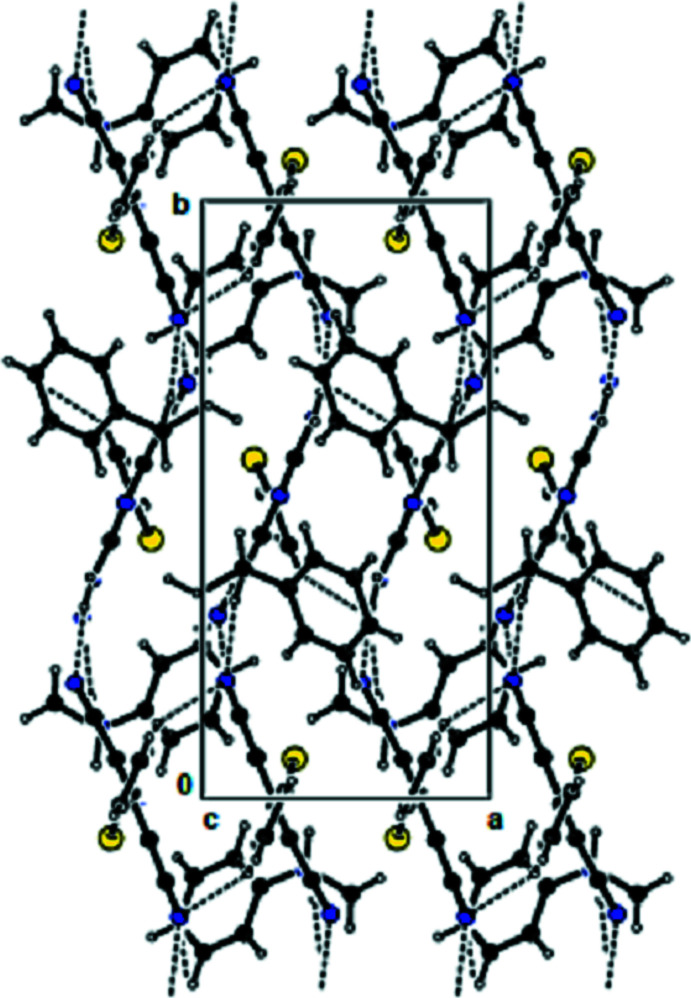
View of the mol­ecular packing along the *c* axis. Hydrogen bonds are shown as dashed lines.

**Figure 6:**
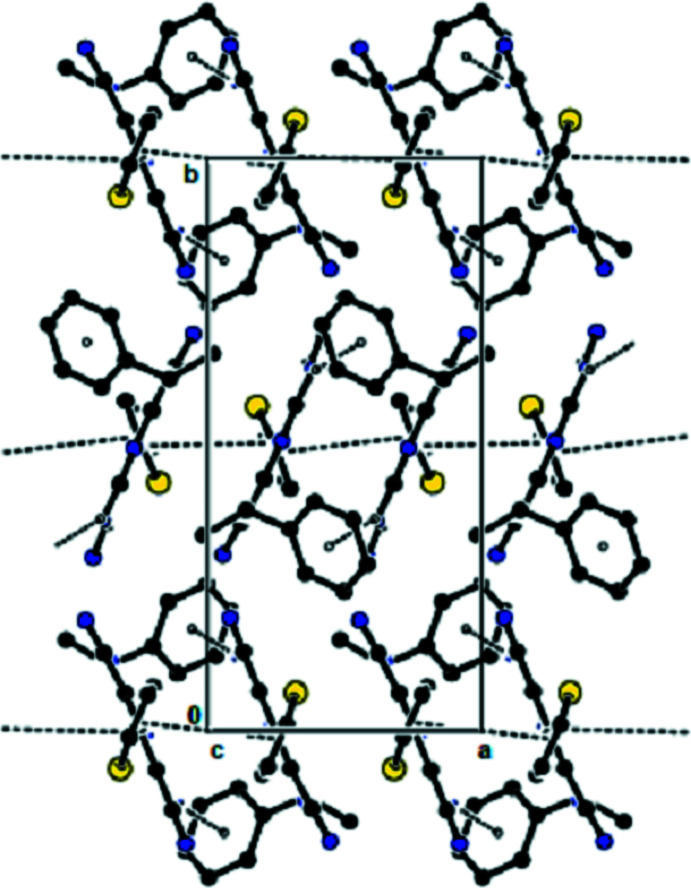
View of the mol­ecular packing along the *c* axis. N—H⋯π inter­actions and π–π stacking inter­actions are shown as dashed lines.

**Table 1:** Hydrogen-bond geometry (Å, °) *Cg*4 is the centroid of the C7–C12 ring.

*D*—H⋯*A*	*D*—H	H⋯*A*	*D*⋯*A*	*D*—H⋯*A*
N2—H2⋯N19^i^	0.91 (3)	2.28 (3)	3.152 (3)	163 (3)
N6—H6*B*⋯N14^ii^	0.89 (3)	2.17 (4)	3.033 (3)	164 (3)
N6—H6*A*⋯*Cg*4^iii^	0.91 (3)	2.62 (4)	3.405 (2)	145 (3)

Symmetry codes: (i) 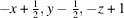 ; (ii) 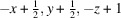 ; (iii) 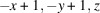 .

**Table 2:** Summary of short inter­atomic contacts (Å) in the title compound

Contact	Distance	Symmetry operation
H13*A*⋯H6*A*	2.36	−*x*, 1 − *y*, *z*
H6*B*⋯N14	2.18	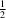 − *x*, 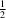 + *y*, 1 − *z*
H16⋯N19	2.56	1 − *x*, 1 − *y*, *z*
C17⋯H9	2.86	− 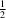 + *x*, 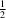 − *y*, 1 − *z*
C10⋯C13	3.58	1 + *x*, *y*, *z*
H12⋯H18*A*	2.31	*x*, *y*, 1 + *z*
H18⋯H11	2.34	1 − *x*, 1 − *y*, −1 + *z*

**Table 3:** Experimental details

Crystal data	
Chemical formula	C_19_H_15_N_5_S
*M*_r_	345.42
Crystal system, space group	Orthorhombic, *P*2_1_2_1_2
Temperature (K)	100
*a*, *b*, *c* (Å)	7.89079 (13), 16.4990 (3), 13.1394 (3)
*V* (Å^3^)	1710.62 (6)
*Z*	4
Radiation type	Cu *K*α
μ (mm^−1^)	1.77
Crystal size (mm)	0.40 × 0.04 × 0.03

Data collection	
Diffractometer	XtaLAB Synergy, Dualflex, HyPix
Absorption correction	Gaussian (*CrysAlis PRO*; Rigaku OD, 2022 ▸)
*T*_min_, *T*_max_	0.532, 0.939
No. of measured, independent and observed [*I* > 2σ(*I*)] reflections	26907, 3713, 3612
*R*_int_	0.044
(sin θ/λ)_max_ (Å^−1^)	0.638

Refinement	
*R*[*F* ^2^ > 2σ(*F* ^2^)], *wR*(*F* ^2^), *S*	0.036, 0.096, 1.05
No. of reflections	3713
No. of parameters	276
No. of restraints	12
H-atom treatment	H atoms treated by a mixture of independent and constrained refinement
Δρ_max_, Δρ_min_ (e Å^−3^)	0.15, −0.25
Absolute structure	Refined as an inversion twin
Absolute structure parameter	0.13 (3)

Computer programs: *CrysAlis PRO* (Rigaku OD, 2022 ▸), *SHELXT2014/5* (Sheldrick, 2015*a* ▸), *SHELXL2018/3* (Sheldrick, 2015*b* ▸), *ORTEP-3 for Windows* (Farrugia, 2012 ▸) and *PLATON* (Spek, 2020 ▸).

## supplementary crystallographic information

### Crystal data

**Table:**

C_19_H_15_N_5_S	*D*_x_ = 1.341 Mg m^−^^3^
*M**_r_* = 345.42	Cu *K*α radiation, λ = 1.54184 Å
Orthorhombic, *P*2_1_2_1_2	Cell parameters from 17132 reflections
*a* = 7.89079 (13) Å	θ = 3.4–79.2°
*b* = 16.4990 (3) Å	µ = 1.77 mm^−^^1^
*c* = 13.1394 (3) Å	*T* = 100 K
*V* = 1710.62 (6) Å^3^	Needle, colourless
*Z* = 4	0.40 × 0.04 × 0.03 mm
*F*(000) = 720	

### Data collection

**Table:**

XtaLAB Synergy, Dualflex, HyPix diffractometer	3612 reflections with *I* > 2σ(*I*)
Radiation source: micro-focus sealed X-ray tube	*R*_int_ = 0.044
φ and ω scans	θ_max_ = 79.7°, θ_min_ = 3.4°
Absorption correction: gaussian (CrysAlisPro; Rigaku OD, 2022)	*h* = −10→9
*T*_min_ = 0.532, *T*_max_ = 0.939	*k* = −21→20
26907 measured reflections	*l* = −16→16
3713 independent reflections	

### Refinement

**Table:**

Refinement on *F*^2^	Secondary atom site location: difference Fourier map
Least-squares matrix: full	Hydrogen site location: mixed
*R*[*F*^2^ > 2σ(*F*^2^)] = 0.036	H atoms treated by a mixture of independent and constrained refinement
*wR*(*F*^2^) = 0.096	*w* = 1/[σ^2^(*F*_o_^2^) + (0.0507*P*)^2^ + 0.5356*P*] where *P* = (*F*_o_^2^ + 2*F*_c_^2^)/3
*S* = 1.05	(Δ/σ)_max_ = 0.001
3713 reflections	Δρ_max_ = 0.15 e Å^−^^3^
276 parameters	Δρ_min_ = −0.25 e Å^−^^3^
12 restraints	Absolute structure: Refined as an inversion twin
Primary atom site location: difference Fourier map	Absolute structure parameter: 0.13 (3)

### Special details

**Table:**

Experimental. CrysAlisPro 1.171.41.123a (Rigaku OD, 2022); Numerical absorption correction based on Gaussian integration over a multifaceted crystal model; Empirical absorption correction using spherical harmonics implemented in SCALE3 ABSPACK scaling algorithm.
Geometry. All esds (except the esd in the dihedral angle between two l.s. planes) are estimated using the full covariance matrix. The cell esds are taken into account individually in the estimation of esds in distances, angles and torsion angles; correlations between esds in cell parameters are only used when they are defined by crystal symmetry. An approximate (isotropic) treatment of cell esds is used for estimating esds involving l.s. planes.
Refinement. Refined as a 2-component inversion twin.

### Fractional atomic coordinates and isotropic or equivalent isotropic displacement parameters (Å^2^)

**Table:**

	*x*	*y*	*z*	*U*_iso_*/*U*_eq_	Occ. (<1)
S1	0.1793 (3)	0.56636 (9)	0.24471 (14)	0.0306 (3)	0.6
S1A	0.3236 (6)	0.42012 (16)	0.2521 (3)	0.0254 (6)	0.4
N1	0.2686 (2)	0.50614 (12)	0.64994 (15)	0.0250 (4)	
C1	0.1399 (3)	0.38542 (14)	0.77369 (17)	0.0270 (5)	
H1	0.1288	0.4439	0.7928	0.032*	
C2	0.2054 (3)	0.44038 (13)	0.60337 (17)	0.0239 (4)	
N2	0.1451 (2)	0.38039 (12)	0.66239 (15)	0.0263 (4)	
H2	0.112 (4)	0.332 (2)	0.636 (2)	0.032*	
C3	0.1980 (3)	0.43395 (13)	0.49462 (17)	0.0237 (4)	
C4	0.2528 (2)	0.49903 (13)	0.43479 (15)	0.0236 (4)	
C5	0.3127 (3)	0.56866 (13)	0.48450 (17)	0.0244 (4)	
C6	0.3206 (3)	0.56878 (12)	0.59318 (17)	0.0242 (4)	
N6	0.3851 (3)	0.63253 (13)	0.64322 (17)	0.0288 (4)	
H6A	0.392 (4)	0.6297 (19)	0.712 (3)	0.035*	
H6B	0.413 (4)	0.679 (2)	0.615 (2)	0.035*	
C7	0.2992 (3)	0.35278 (14)	0.82352 (17)	0.0255 (4)	
C8	0.3550 (3)	0.27466 (15)	0.80460 (19)	0.0313 (5)	
H8	0.2958	0.2417	0.7571	0.038*	
C9	0.4974 (3)	0.24370 (16)	0.8545 (2)	0.0352 (5)	
H9	0.5354	0.1903	0.8402	0.042*	
C10	0.5827 (3)	0.29053 (17)	0.9245 (2)	0.0352 (5)	
H10	0.6777	0.2691	0.9599	0.042*	
C11	0.5292 (3)	0.3690 (2)	0.9429 (2)	0.0429 (6)	
H11	0.5883	0.4019	0.9904	0.051*	
C12	0.3887 (3)	0.39989 (17)	0.8918 (2)	0.0361 (6)	
H12	0.3540	0.4541	0.9041	0.043*	
C13	−0.0172 (3)	0.34112 (18)	0.8124 (2)	0.0361 (6)	
H13A	−0.1190	0.3670	0.7847	0.054*	
H13B	−0.0203	0.3435	0.8869	0.054*	
H13C	−0.0133	0.2844	0.7904	0.054*	
C14	0.1206 (3)	0.36403 (14)	0.45060 (17)	0.0257 (4)	
N14	0.0540 (3)	0.30718 (12)	0.41871 (16)	0.0310 (4)	
C15	0.251 (3)	0.4906 (6)	0.3229 (3)	0.029 (3)	0.6
C16	0.3006 (16)	0.4236 (4)	0.2681 (5)	0.025 (2)	0.6
H16	0.3422	0.3748	0.2974	0.030*	0.6
C17	0.2816 (10)	0.4371 (4)	0.1618 (5)	0.0346 (15)	0.6
H17	0.3135	0.3981	0.1121	0.042*	0.6
C18	0.2137 (10)	0.5107 (4)	0.1371 (5)	0.0366 (17)	0.6
H18	0.1893	0.5284	0.0698	0.044*	0.6
C15A	0.246 (4)	0.4989 (10)	0.3229 (3)	0.024 (4)	0.4
C16A	0.180 (2)	0.5595 (5)	0.2643 (5)	0.025 (2)	0.4
H16A	0.1294	0.6078	0.2899	0.030*	0.4
C17A	0.1963 (15)	0.5398 (6)	0.1596 (6)	0.029 (2)	0.4
H17A	0.1595	0.5750	0.1069	0.035*	0.4
C18A	0.2696 (14)	0.4661 (7)	0.1404 (6)	0.032 (2)	0.4
H18A	0.2877	0.4439	0.0746	0.038*	0.4
C19	0.3707 (3)	0.63853 (14)	0.43145 (18)	0.0256 (4)	
N19	0.4186 (3)	0.69712 (13)	0.39337 (16)	0.0298 (4)	

### Atomic displacement parameters (Å^2^)

**Table:**

	*U*^11^	*U*^22^	*U*^33^	*U*^12^	*U*^13^	*U*^23^
S1	0.0317 (6)	0.0288 (5)	0.0314 (6)	−0.0050 (5)	−0.0049 (7)	0.0067 (5)
S1A	0.0253 (13)	0.0248 (10)	0.0261 (9)	0.0020 (6)	0.0027 (10)	−0.0027 (8)
N1	0.0238 (8)	0.0243 (9)	0.0269 (9)	−0.0001 (7)	−0.0007 (7)	−0.0006 (7)
C1	0.0261 (10)	0.0271 (10)	0.0279 (11)	0.0024 (8)	0.0027 (9)	−0.0004 (8)
C2	0.0183 (9)	0.0223 (9)	0.0311 (11)	0.0022 (8)	0.0001 (8)	0.0025 (8)
N2	0.0276 (9)	0.0248 (9)	0.0266 (9)	−0.0007 (7)	0.0000 (7)	0.0024 (7)
C3	0.0199 (9)	0.0236 (10)	0.0276 (10)	0.0018 (9)	−0.0015 (8)	0.0009 (8)
C4	0.0157 (9)	0.0249 (11)	0.0303 (11)	0.0022 (8)	−0.0005 (7)	0.0013 (9)
C5	0.0190 (9)	0.0246 (10)	0.0298 (10)	0.0012 (9)	0.0002 (8)	0.0012 (8)
C6	0.0190 (9)	0.0221 (10)	0.0314 (11)	0.0014 (8)	−0.0005 (8)	−0.0016 (8)
N6	0.0314 (9)	0.0257 (10)	0.0293 (10)	−0.0028 (8)	−0.0022 (8)	−0.0013 (8)
C7	0.0234 (10)	0.0289 (11)	0.0242 (9)	−0.0018 (8)	0.0052 (8)	0.0007 (8)
C8	0.0296 (11)	0.0290 (11)	0.0355 (12)	−0.0022 (9)	−0.0070 (9)	−0.0009 (9)
C9	0.0301 (11)	0.0311 (12)	0.0443 (13)	0.0014 (10)	−0.0047 (11)	0.0011 (11)
C10	0.0246 (10)	0.0491 (15)	0.0319 (11)	−0.0009 (10)	−0.0018 (9)	0.0025 (11)
C11	0.0339 (13)	0.0532 (16)	0.0415 (14)	−0.0033 (12)	−0.0083 (11)	−0.0148 (13)
C12	0.0335 (12)	0.0384 (14)	0.0364 (12)	0.0008 (10)	−0.0007 (10)	−0.0108 (11)
C13	0.0260 (11)	0.0479 (15)	0.0344 (12)	0.0015 (11)	0.0037 (10)	0.0083 (11)
C14	0.0245 (9)	0.0252 (10)	0.0273 (10)	0.0023 (8)	−0.0004 (8)	0.0028 (9)
N14	0.0334 (10)	0.0264 (10)	0.0331 (10)	−0.0023 (8)	−0.0034 (8)	0.0012 (8)
C15	0.022 (6)	0.027 (4)	0.036 (6)	−0.011 (3)	−0.004 (4)	0.009 (3)
C16	0.023 (3)	0.033 (3)	0.020 (3)	0.0021 (19)	0.003 (2)	−0.0031 (17)
C17	0.027 (3)	0.049 (4)	0.028 (3)	−0.006 (3)	−0.001 (2)	−0.003 (3)
C18	0.029 (3)	0.061 (6)	0.020 (3)	−0.016 (4)	0.001 (3)	0.004 (3)
C15A	0.017 (7)	0.036 (6)	0.020 (6)	0.001 (6)	0.004 (6)	−0.005 (5)
C16A	0.023 (3)	0.033 (3)	0.020 (3)	0.0021 (19)	0.003 (2)	−0.0031 (17)
C17A	0.027 (3)	0.048 (6)	0.012 (4)	0.000 (4)	0.003 (3)	−0.001 (3)
C18A	0.022 (4)	0.053 (8)	0.020 (4)	0.011 (5)	−0.001 (3)	0.005 (5)
C19	0.0224 (9)	0.0251 (10)	0.0293 (10)	0.0005 (8)	−0.0009 (9)	−0.0030 (9)
N19	0.0300 (9)	0.0265 (10)	0.0331 (10)	−0.0034 (8)	0.0004 (8)	−0.0006 (8)

### Geometric parameters (Å, º)

**Table:**

S1—C18	1.708 (4)	C8—H8	0.9500
S1—C15	1.713 (4)	C9—C10	1.377 (4)
S1A—C18A	1.707 (4)	C9—H9	0.9500
S1A—C15A	1.711 (4)	C10—C11	1.384 (4)
N1—C6	1.339 (3)	C10—H10	0.9500
N1—C2	1.342 (3)	C11—C12	1.392 (4)
C1—N2	1.465 (3)	C11—H11	0.9500
C1—C7	1.516 (3)	C12—H12	0.9500
C1—C13	1.526 (3)	C13—H13A	0.9800
C1—H1	1.0000	C13—H13B	0.9800
C2—N2	1.344 (3)	C13—H13C	0.9800
C2—C3	1.434 (3)	C14—N14	1.154 (3)
N2—H2	0.91 (3)	C15—C16	1.377 (4)
C3—C4	1.399 (3)	C16—C17	1.423 (4)
C3—C14	1.428 (3)	C16—H16	0.9500
C4—C5	1.404 (3)	C17—C18	1.368 (9)
C4—C15A	1.472 (4)	C17—H17	0.9500
C4—C15	1.477 (3)	C18—H18	0.9500
C5—C19	1.423 (3)	C15A—C16A	1.368 (4)
C5—C6	1.429 (3)	C16A—C17A	1.420 (4)
C6—N6	1.341 (3)	C16A—H16A	0.9500
N6—H6A	0.91 (3)	C17A—C18A	1.369 (13)
N6—H6B	0.89 (3)	C17A—H17A	0.9500
C7—C12	1.382 (3)	C18A—H18A	0.9500
C7—C8	1.384 (3)	C19—N19	1.152 (3)
C8—C9	1.398 (3)		
			
C18—S1—C15	93.0 (4)	C9—C10—C11	119.5 (2)
C18A—S1A—C15A	92.3 (5)	C9—C10—H10	120.2
C6—N1—C2	118.95 (19)	C11—C10—H10	120.2
N2—C1—C7	112.82 (18)	C10—C11—C12	120.1 (2)
N2—C1—C13	109.13 (19)	C10—C11—H11	119.9
C7—C1—C13	111.07 (19)	C12—C11—H11	119.9
N2—C1—H1	107.9	C7—C12—C11	121.0 (3)
C7—C1—H1	107.9	C7—C12—H12	119.5
C13—C1—H1	107.9	C11—C12—H12	119.5
N1—C2—N2	117.6 (2)	C1—C13—H13A	109.5
N1—C2—C3	122.0 (2)	C1—C13—H13B	109.5
N2—C2—C3	120.4 (2)	H13A—C13—H13B	109.5
C2—N2—C1	122.9 (2)	C1—C13—H13C	109.5
C2—N2—H2	122 (2)	H13A—C13—H13C	109.5
C1—N2—H2	115 (2)	H13B—C13—H13C	109.5
C4—C3—C14	121.65 (19)	N14—C14—C3	177.1 (2)
C4—C3—C2	119.4 (2)	C16—C15—C4	126.3 (4)
C14—C3—C2	118.7 (2)	C16—C15—S1	111.5 (3)
C3—C4—C5	118.08 (19)	C4—C15—S1	122.2 (3)
C3—C4—C15A	123.3 (11)	C15—C16—C17	110.9 (5)
C5—C4—C15A	118.6 (11)	C15—C16—H16	124.5
C3—C4—C15	118.9 (7)	C17—C16—H16	124.5
C5—C4—C15	123.0 (7)	C18—C17—C16	114.4 (6)
C4—C5—C19	122.9 (2)	C18—C17—H17	122.8
C4—C5—C6	118.7 (2)	C16—C17—H17	122.8
C19—C5—C6	118.3 (2)	C17—C18—S1	110.1 (6)
N1—C6—N6	116.7 (2)	C17—C18—H18	125.0
N1—C6—C5	122.8 (2)	S1—C18—H18	125.0
N6—C6—C5	120.5 (2)	C16A—C15A—C4	125.1 (5)
C6—N6—H6A	118 (2)	C16A—C15A—S1A	112.8 (3)
C6—N6—H6B	125 (2)	C4—C15A—S1A	122.1 (4)
H6A—N6—H6B	117 (3)	C15A—C16A—C17A	109.9 (6)
C12—C7—C8	118.5 (2)	C15A—C16A—H16A	125.0
C12—C7—C1	120.3 (2)	C17A—C16A—H16A	125.0
C8—C7—C1	121.1 (2)	C18A—C17A—C16A	115.0 (9)
C7—C8—C9	120.8 (2)	C18A—C17A—H17A	122.5
C7—C8—H8	119.6	C16A—C17A—H17A	122.5
C9—C8—H8	119.6	C17A—C18A—S1A	110.0 (8)
C10—C9—C8	120.1 (2)	C17A—C18A—H18A	125.0
C10—C9—H9	120.0	S1A—C18A—H18A	125.0
C8—C9—H9	120.0	N19—C19—C5	176.4 (2)
			
C6—N1—C2—N2	−176.22 (19)	C1—C7—C8—C9	−176.9 (2)
C6—N1—C2—C3	2.5 (3)	C7—C8—C9—C10	0.9 (4)
N1—C2—N2—C1	2.4 (3)	C8—C9—C10—C11	−1.7 (4)
C3—C2—N2—C1	−176.30 (19)	C9—C10—C11—C12	0.8 (4)
C7—C1—N2—C2	−90.5 (2)	C8—C7—C12—C11	−1.8 (4)
C13—C1—N2—C2	145.6 (2)	C1—C7—C12—C11	176.0 (2)
N1—C2—C3—C4	−2.2 (3)	C10—C11—C12—C7	1.0 (4)
N2—C2—C3—C4	176.47 (18)	C3—C4—C15—C16	−42 (2)
N1—C2—C3—C14	−176.64 (19)	C5—C4—C15—C16	135.4 (17)
N2—C2—C3—C14	2.0 (3)	C15A—C4—C15—C16	173 (27)
C14—C3—C4—C5	174.0 (2)	C3—C4—C15—S1	136.5 (12)
C2—C3—C4—C5	−0.3 (3)	C5—C4—C15—S1	−46 (2)
C14—C3—C4—C15A	−4.1 (12)	C15A—C4—C15—S1	−8 (23)
C2—C3—C4—C15A	−178.4 (11)	C18—S1—C15—C16	0.1 (16)
C14—C3—C4—C15	−8.0 (9)	C18—S1—C15—C4	−179.0 (17)
C2—C3—C4—C15	177.7 (8)	C4—C15—C16—C17	−179.8 (17)
C3—C4—C5—C19	−179.6 (2)	S1—C15—C16—C17	1 (2)
C15A—C4—C5—C19	−1.4 (11)	C15—C16—C17—C18	−2.2 (17)
C15—C4—C5—C19	2.4 (9)	C16—C17—C18—S1	2.3 (10)
C3—C4—C5—C6	2.2 (3)	C15—S1—C18—C17	−1.3 (10)
C15A—C4—C5—C6	−179.5 (11)	C3—C4—C15A—C16A	130 (2)
C15—C4—C5—C6	−175.7 (8)	C5—C4—C15A—C16A	−48 (4)
C2—N1—C6—N6	−179.01 (19)	C15—C4—C15A—C16A	168 (28)
C2—N1—C6—C5	−0.4 (3)	C3—C4—C15A—S1A	−50 (3)
C4—C5—C6—N1	−2.0 (3)	C5—C4—C15A—S1A	132.2 (18)
C19—C5—C6—N1	179.74 (19)	C15—C4—C15A—S1A	−12 (23)
C4—C5—C6—N6	176.58 (19)	C18A—S1A—C15A—C16A	0 (2)
C19—C5—C6—N6	−1.6 (3)	C18A—S1A—C15A—C4	−180 (2)
N2—C1—C7—C12	126.2 (2)	C4—C15A—C16A—C17A	179 (2)
C13—C1—C7—C12	−110.9 (3)	S1A—C15A—C16A—C17A	−1 (3)
N2—C1—C7—C8	−56.0 (3)	C15A—C16A—C17A—C18A	1 (2)
C13—C1—C7—C8	66.9 (3)	C16A—C17A—C18A—S1A	−1.2 (15)
C12—C7—C8—C9	0.9 (4)	C15A—S1A—C18A—C17A	0.6 (15)

### Hydrogen-bond geometry (Å, º)

*Cg*4 is the centroid of the C7–C12 ring.

**Table:**

*D*—H···*A*	*D*—H	H···*A*	*D*···*A*	*D*—H···*A*
N2—H2···N19^i^	0.91 (3)	2.28 (3)	3.152 (3)	163 (3)
N6—H6*B*···N14^ii^	0.89 (3)	2.17 (4)	3.033 (3)	164 (3)
N6—H6*A*···*Cg*4^iii^	0.91 (3)	2.62 (4)	3.405 (2)	145 (3)

Symmetry codes: (i) −*x*+1/2, *y*−1/2, −*z*+1; (ii) −*x*+1/2, *y*+1/2, −*z*+1; (iii) −*x*+1, −*y*+1, *z*.

## References

[bb1] Bernstein, J., Davis, R. E., Shimoni, L. & Chang, N.-L. (1995). *Angew. Chem. Int. Ed. Engl.* **34**, 1555–1573.

[bb2] Çelik, M. S., Çetinus, A., Yenidünya, A. F., Çetinkaya, S. & Tüzün, B. (2023). *J. Mol. Struct.* **1272**, 134158.

[bb3] Chalkha, M., Ameziane el Hassani, A., Nakkabi, A., Tüzün, B., Bakhouch, M., Benjelloun, A. T., Sfaira, M., Saadi, M., Ammari, L. E. & Yazidi, M. E. (2023). *J. Mol. Struct.* **1273**, 134255.

[bb4] Cocco, M. T., Congiu, C., Lilliu, V. & Onnis, V. (2005). *Eur. J. Med. Chem.* **40**, 1365–1372. 10.1016/j.ejmech.2005.07.005 16137795

[bb5] Farrugia, L. J. (2012). *J. Appl. Cryst.* **45**, 849–854.

[bb6] Groom, C. R., Bruno, I. J., Lightfoot, M. P. & Ward, S. C. (2016). *Acta Cryst.* B**72**, 171–179. 10.1107/S2052520616003954 PMC4822653 27048719

[bb7] Gurbanov, A. V., Kuznetsov, M. L., Mahmudov, K. T., Pombeiro, A. J. L. & Resnati, G. (2020). *Chem. Eur. J.* **26**, 14833–14837. 10.1002/chem.202002518 32567710

[bb8] Naghiyev, F. N., Akkurt, M., Askerov, R. K., Mamedov, I. G., Rzayev, R. M., Chyrka, T. & Maharramov, A. M. (2020). *Acta Cryst.* E**76**, 720–723. 10.1107/S2056989020005381 PMC7199244 32431939

[bb9] Naghiyev, F. N., Khrustalev, V. N., Novikov, A. P., Akkurt, M., Rzayev, R. M., Akobirshoeva, A. A. & Mamedov, I. G. (2022). *Acta Cryst.* E**78**, 554–558. 10.1107/S2056989022004741 PMC9431780 36072149

[bb10] Naghiyev, F. N., Tereshina, T. A., Khrustalev, V. N., Akkurt, M., Rzayev, R. M., Akobirshoeva, A. A. & Mamedov, İ. G. (2021). *Acta Cryst.* E**77**, 516–521. 10.1107/S2056989021003583 PMC8100256 34026256

[bb11] Poustforoosh, A., Hashemipour, H., Tüzün, B., Azadpour, M., Faramarz, S., Pardakhty, A., Mehrabani, M. & Nematollahi, M. H. (2022). *Curr. Microbiol.* **79**, 241. 10.1007/s00284-022-02921-6 PMC9258457 35792936

[bb12] Rigaku OD (2022). *CrysAlis PRO*. Rigaku Oxford Diffraction, Yarnton, England.

[bb13] Sheldrick, G. M. (2015*a*). *Acta Cryst.* A**71**, 3–8.

[bb14] Sheldrick, G. M. (2015*b*). *Acta Cryst.* C**71**, 3–8.

[bb15] Spackman, P. R., Turner, M. J., McKinnon, J. J., Wolff, S. K., Grimwood, D. J., Jayatilaka, D. & Spackman, M. A. (2021). *J. Appl. Cryst.* **54**, 1006–1011. 10.1107/S1600576721002910 PMC8202033 34188619

[bb16] Spek, A. L. (2020). *Acta Cryst.* E**76**, 1–11. 10.1107/S2056989019016244 PMC6944088 31921444

[bb17] Tapera, M., Kekeçmuhammed, H., Tüzün, B., Sarıpınar, E., Koçyiğit, M., Yıldırım, E., Doğan, M. & Zorlu, Y. (2022). *J. Mol. Struct.* **1269**, 133816.

[bb18] Vishnupriya, R., Suresh, J., Bharkavi, S., Perumal, S. & Lakshman, P. L. N. (2014*a*). *Acta Cryst.* E**70**, o968–o969. 10.1107/S1600536814017188 PMC4186133 25309284

[bb19] Vishnupriya, R., Suresh, J., Gunasekaran, P., Perumal, S. & Lakshman, P. L. N. (2014*b*). *Acta Cryst.* E**70**, o978. 10.1107/S1600536814017693 PMC4186124 25309290

[bb20] Vishnupriya, R., Suresh, J., Sakthi, M., Perumal, S. & Lakshman, P. L. N. (2014*c*). *Acta Cryst.* E**70**, o1120–o1121. 10.1107/S1600536814020170 PMC4257225 25484706

[bb21] Vu Quoc, T., Tran Thi Thuy, D., Phung Ngoc, T., Vu Quoc, M., Nguyen, H., Duong Khanh, L., Tu Quang, A. & Van Meervelt, L. (2019). *Acta Cryst.* E**75**, 1861–1865. 10.1107/S2056989019015081 PMC6895956 31871746

[bb22] Zhang, X., Tao, F., Cui, T., Luo, C., Zhou, Z., Huang, Y., Tan, L., Peng, W. & Wu, C. (2022). *Molecules*, **27**, 7187. 10.3390/molecules27217187 PMC9656638 36364013

[bb23] Zubkov, F. I., Mertsalov, D. F., Zaytsev, V. P., Varlamov, A. V., Gurbanov, A. V., Dorovatovskii, P. V., Timofeeva, T. V., Khrustalev, V. N. & Mahmudov, K. T. (2018). *J. Mol. Liq.* **249**, 949–952.

